# Developing a ‘personalome’ for precision medicine: emerging methods that compute interpretable effect sizes from single-subject transcriptomes

**DOI:** 10.1093/bib/bbx149

**Published:** 2017-12-18

**Authors:** Francesca Vitali, Qike Li, A Grant Schissler, Joanne Berghout, Colleen Kenost, Yves A Lussier

**Affiliations:** 1 BIO5 Institute, University of Arizona, Tucson, AZ, USA; 2 University of Nevada, Reno

**Keywords:** single-subject studies, personalome, precision medicine, n-of-1

## Abstract

The development of computational methods capable of analyzing -omics data at the individual level is critical for the success of precision medicine. Although unprecedented opportunities now exist to gather data on an individual’s -omics profile (‘personalome’), interpreting and extracting meaningful information from single-subject -omics remain underdeveloped, particularly for quantitative non-sequence measurements, including complete transcriptome or proteome expression and metabolite abundance. Conventional bioinformatics approaches have largely been designed for making population-level inferences about ‘average’ disease processes; thus, they may not adequately capture and describe individual variability. Novel approaches intended to exploit a variety of -omics data are required for identifying individualized signals for meaningful interpretation. In this review—intended for biomedical researchers, computational biologists and bioinformaticians—we survey emerging computational and translational informatics methods capable of constructing a single subject's ‘personalome’ for predicting clinical outcomes or therapeutic responses, with an emphasis on methods that provide interpretable readouts. Key points: (i) the single-subject analytics of the transcriptome shows the greatest development to date and, (ii) the methods were all validated in simulations, cross-validations or independent retrospective data sets. This survey uncovers a growing field that offers numerous opportunities for the development of novel validation methods and opens the door for future studies focusing on the interpretation of comprehensive ‘personalomes’ through the integration of multiple -omics, providing valuable insights into individual patient outcomes and treatments.

## Introduction

The arrival of precision medicine has led to a more individual-based view of diseases, with characteristics of single subjects being central to the prediction of clinical outcomes and prescription of tailored treatments. This concept is not new; in fact, evidence-based clinical practice guidelines [1] stratify treatments according to some patient characteristics (e.g. gender, ancestry, age, family history, some laboratory test results). However, precision medicine differs from the traditional medical approach, as it seeks to leverage not only clinical variables and clinician-selected genetic tests but also broad and data-intensive molecular and general -omics profiles of a patient [2]. These large and heterogeneous data cannot be interpreted directly by medical practitioners and require an automatic procedure for extracting relevant knowledge before incorporation into clinical practice. Therefore, it is fundamental to develop computational methods aimed at analyzing these data at the individual level.

Current approaches aimed at analyzing disease or other biological processes, therapeutic efficacy and -omic data still leverage well-established cohort-based population analyses such as case-control studies [e.g. gene expression classifiers (GExpCs)], observational trials or controlled intervention trials. These large cohort/group approaches place emphasis on the group average rather than individual participants; though this group average may not represent any actual individual’s personal profile, let alone be meaningful to understanding the profile of a given specific patient. On the other hand, the framework of N-of-1 trials has been applied to repeated measures of a single analyte for over two decades [3]. This approach is based on the collection of various relevant data for one person as frequently as possible [4]. In this way, novel strategies can be explored to compare different treatments of the same person. Moreover, by looking at commonalities across multiple N-of-1 studies collecting the same type of data, it is possible to estimate the efficacy of an intervention in a specific subset population (i.e. people sharing a particular genetic profile). N-of-1 trials demonstrated their power to evaluate treatment effectiveness in a single subject for one variable [5], but proposed approaches for one analyte do not scale for -omics legion-size data sets.

Although we now have an unprecedented technical opportunity to gather data relating to an individual’s -omic profile, bioinformatics tools to understand these data comprehensively, and at the individual level, remain underdeveloped. Novel approaches for identifying individualized (single-subject)—and not cohort—signals are required for gathering insights into the biology of diseases and healthy states of individuals. This review focuses on computational methods aimed at analyzing quantitative transcriptomic measurements of an individual and the combination of transcriptome with other -omic data.

**Figure 1:**
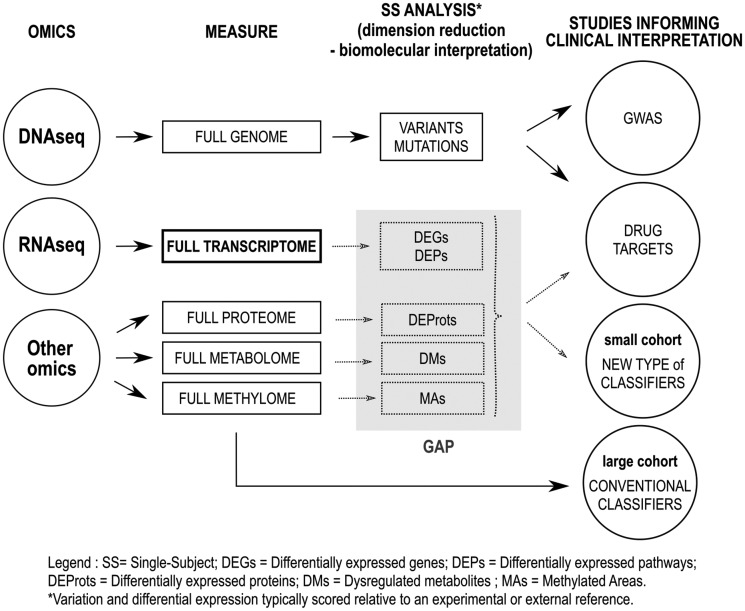
Flow chart of methods designed for clinical interpretation of single-subject -omics. This review addresses the gap of knowledge to compare and contrast single-subject methods designed to reduce the dimension of raw -omics data (left) and to provide a biomolecular interpretation of signals (gray rectangle). For DNA sequencing, variant and mutations calls as well as all functional annotations in single subjects (e.g. missense mutation) already bridge this gap. However, this intermediate step is often omitted for other molecules of life, such as mRNAs, miRNAs, proteins, methylated DNA regions and metabolites (carbohydrates and lipids). This review focuses on single-subject methods that analyze transcriptome data. ‘Clinical applications’ section provides emerging evidence that the newly available, unbiased SSA of the transcriptome enable innovative types of studies to investigate their clinical utility by addressing the gap of biomolecular interpretation of raw -omics signals. Among possible studies, we demonstrate that -omics clinical prediction classifiers that operate directly at the -omics scale may be redesigned for the parsimonious transformed signal of single-subject studies for improved clinical utility.

In this review, we define the personalome as an interpretable personal molecular mechanism profile of an individual derived from one or more scales of -omic data, especially when designed to enable precision medicine. ‘Personal -omics’ means the -omics measures of a single subject. Molecular mechanisms are any molecular functions or biological processes such as a missense mutation in DNA, or a differentially expressed pathway (DEP) at the transcriptome or proteome. To be considered interpretable at the molecular mechanism, the raw -omics profile must have been subjected to analyses performing (i) dimension reduction and (ii) biomolecular interpretation of the mechanisms involved in molecules of life (Figure 1). For example, full genomes are reduced to variant and mutation calls through analyses against a reference genome, or in the case of cancer, by also comparing paired cancer and unaffected tissue to determine somatic versus germline mutations. Here, we show how differentially expressed molecules of life and pathways can be unveiled in a single subject through the analysis of transcriptome data.

We surveyed emerging novel computational biology, biostatistical and translational informatics methods that construct a single subject’s personalome by analyzing transcriptome data to predict outcomes or therapeutic responses without requiring the large cohort needed for conventional approaches.

**Figure 2:**
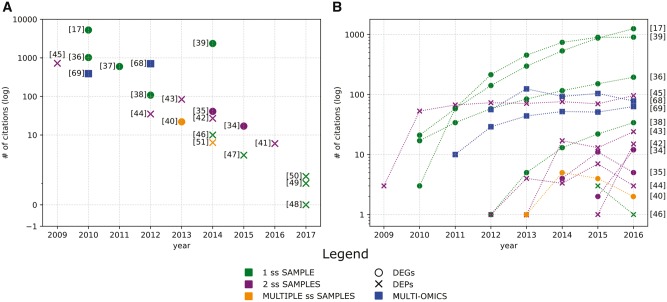
SSA studies included in this review. (**A**) Each numbered point represents a publication plotted by year of publication and the relative number of citations (in log2 scale). Numbers correspond to the publication in this article’s reference list, colors indicate the type of input required, i.e. one single-subject sample (1 ss SAMPLE—green), two paired single-subject sample (2 ss SAMPLES—purple) or if the method requires the collection of multiple samples from the same subject (multiple ss SAMPLES—orange). The shapes represent the type of output provided by the selected studies, i.e. DEGs—circle, DEPs—X. Finally, blue squares indicate methods based on the integration of transcriptome data with other - omics. (**B**) Number of citations over time starting from the publication year for the single-subject studies analyzing transcriptome data. Color and shape codification is the same as for the (**A**).

Our review methodology is detailed in the Supplementary Material S1. Particular emphasis is placed on those methods that provide clinically interpretable readouts rather than simple categorical classification, as the latter are known to be difficult to reproduce across data sets and contain noisy, incidental and passenger variation [6–9]. The papers and methods selected for review reflect the authors' views and are not intended to provide an exhaustive search. Figure 2A depicts all considered publications by year of publication and number of citations, and the studies are shown with different colors and shapes according to the type of required data input and output, respectively. Figure 2B shows the number of citations over time.

The review is divided according to the type of data inputs in the methods (i.e. transcriptome and integrated -omics). A review of the validations of all methods follows, and finally, we discuss and conclude with the broad challenges, the applications and the opportunities in developing a personalome for precision medicine, i.e. how the single-subject analyses (SSAs) of -omics data can bring novel insights in disease mechanisms specific of a patient and unveil potential patient-specific treatments. A table of content for the review is provided in Table 1.

**Table 1:** Table of content of the review

Section	Pages
Transcriptome	p. 2
Cross-subject transcriptome analyses	p. 4
Single-subject transcriptome analyses	p. 4
DEGs identification in single-subjects	p. 7
DEPs identification in single-subjects	p. 8
Longitudinal time series analyses of transcriptome	p. 10
Single-subject transcriptome integrated with other ‐omics	p. 11
Validation of single-subject methods	p. 12
Clinical applications	p. 12
Perspective and conclusion	p. 13

## Transcriptome

Transcriptome analysis aims to interpret the quantification of transcribed genetic material, including both coding and noncoding RNA. Different from DNA, which is relatively static, analyses of the transcriptome capture the collective impact of tissue type, sequence variation, regulation, environment, external stimulation (e.g. drug treatments) and interactions between them. High-throughput technologies, such as microarray and RNA sequencing (RNA-Seq), are capable of assessing transcript expression at genome-scale for an individual sample, with RNA-Seq providing unbiased detection, broader dynamic range, increased specificity and sensitivity and easier detection of rare and low-abundance transcripts.

The transcriptome provides a snapshot of transcriptional activity under the condition where the RNA was collected, allowing researchers to study the biological impact of certain diseases or effect of treatments [10]. This allows us to better understand general disease mechanisms, discover biomarkers or identify drug targets at the cohort scale when sufficient samples are collected, but also has the power to reveal individual-specific signals, whose detection and analysis through computational methods can lead to far more precise medical understanding and decision-making. Analysis of more than one transcriptome of an individual enables the assessment of personal dynamic changes over time or in response to therapy or other environmental changes. Yet, identifying important individual signals is not a trivial task, as transcript expression variations in a given tissue and time point are further modulated by stochastic variability, cyclic patterns (ex circadian) and platform biases or measurement errors in addition to signals, which are truly relevant to the disease state. The power of the methods reported in this section is that starting from thousands of genes they are able to provide information on the key genes and mechanisms (i.e. pathways) of a disease. This can allow to speed up the planning of future and effective studies.

### Cross-subject transcriptome analyses

Conventional transcriptome analytics require well-powered cohorts of both cases and controls and describe variation in transcriptome when comparing two or more classes with a variety of methods (e.g. *t*-test [11, 12], analysis of variance [13], linear mixed models [14], modeling via the negative binomial distribution [15–17]). These strategies are designed to identify DEGs of ‘average responses across patients’ under particular experimental conditions (e.g. disease versus normal; or predrug and postdrug treatment). To extract more interpretable results, genes detected as differentially expressed are often further categorized according to enrichment or membership in knowledge bases such as curated biological pathways or functional gene sets (e.g. Kyoto Encyclopedia of Genes and Genomes (KEGG) [18]), Gene Ontology (GO) [19]. In this way, DEPs of average responses of patients can be identified, providing a more comprehensible view of the transcriptomic processes under study versus a simple gene list that requires significant gene recognition and subject matter expertise for interpretation. A wide array of studies and tools belong to this category including popular ones as gene set enrichment analysis (GSEA) [20] and DAVID [21]. In general, there are two main strategies to identify DEPs: (i) gene set-centric (GC) and (ii) pathway-centric analyses (PC). The GC approach is generally performed in two steps: first, DEGs are selected and the DEPs are computed by statistically testing the genes against the background. A critical limitation of GC strategy is that the results strongly depend on the DEGs identified in the first step. In fact, small changes in the DEG analysis may lead to the detection of a slightly different DEG list that can result in high changes of the identified DEPs. In addition, the final result is significantly affected by the arbitrary cutoff chosen in the enrichment step, as the majority of statistical test require a *P*-value threshold [22]. Therefore, we are providing the minimum number of genes in each gene set (Figure 5, column ‘Minimum # of transcript per scored gene set’), as methods providing a higher minimal threshold will be less susceptible to this bias. However, another limitation common to all reviewed DEPs is that similar gene sets are not identified as biomolecularly related in the resulting set, though postprocessing methods are available to address it [23–25].

**Table 2:** Additional details on single-subject transcriptome analyses of DEGs

Publication	Name	Description
Wang *et al.* [34]	RankComp	RankComp requires two inputs: (i) a disease sample and (ii) a set of accumulated normal samples, which can be can be accrued during the same experiment or a priori from various external resources. RankComp begins by ranking genes within the samples (both the case and the normal) according to increasing expression values. Next, pairwise rank comparison are performed to identify (a) stable gene pairs, and (b) reversal gene pairs. Stable gene pairs are defined as those with the same ordering in 99% of the accumulated normal samples [expressiongeneA > expressiongeneB] while reversal gene pairs are identified by disruption of that ordering in the disease sample [expressiongeneA < expressiongeneB]. Fisher’s exact test is conducted to test the null hypothesis that the numbers of reversal gene pairs supporting its upregulation or downregulation are equal. This procedure enables extraction of a list of DEGs for a single subject, and interpretable results can be obtained through manual examination or by performing gene set enrichment analyses
Liu *et al.* [35]	DNB	Computational approach based on DNB theory to detect pre-disease states
Wang. *et al.* [36]	DEGseq	DEGseq identifies DEGs using RNA-Seq data collected from a single subject. When replicates are not available, the authors suggest a MA-plot-based method with a random sampling model, which assumes the expression counts follow a binomial distribution. Given the average of log2-transformed expression levels, it approximates the log2 expression fold change by a normal distribution, and then calculates a Z-score based on this distribution. *P*-values are computed based on Z-scores
Tarazona *et al.* [37]	NOISeq	NOISeq is a data-adaptive and nonparametric approach, which has a variant, NOISeq-sim, that works without replicates. NOISeq-sim uses simulated replicates when real replicates do not exist. It simulates replicates under the assumption that gene expression counts follow multinomial distribution in which the probability of each gene corresponds to the probability of a read mapping to that gene. The probability of each gene is estimated by the proportion of its read counts relative to the total number of mapped reads from the only sample under the corresponding experimental condition. With the simulated replicates, NOISeq-sim generates a joint null distribution of fold-changes (M) and absolute differences (D) of the expression counts from the replicates within the same condition. This joint null distribution is then used to assess differential expression by gene‘s (M, D) pair computed between conditions
Feng *et al.* [38]	GFOLD	This method assumes a Poisson distribution (λ) for the gene expression counts and a uniform prior distribution for λ. After computing a posterior distribution of λ for each gene, GFOLD ranks gene expression changes of all genes based on the cth percentile of these posterior distributions, where c is determined by users. In this way, it penalizes genes with low expression levels for their larger variances
Anders *et al.* [39]	DESeq	When neither condition (i.e. affected and control sample) has replicate transcriptomes, DESeq assumes the majority of the genes as non-DEGs and estimates a mean–variance relationship from treating the two samples as if they were replicates [33]
Robinson *et al.* [17]	edgeR	edgeR assumes that RNA-Seq data follow negative binomial distribution for which, given the mean, the variance is determined by a dispersion parameter. When working without replicates, edgeR assigns the same value of the dispersion parameter to all genes and conducts a negative binomial exact test to compute *P*-values. Note that the value of dispersion is predetermined based on investigators' understanding of the biological nature of the samples rather than estimated from data [18]

The PC strategy is a distinct approach to derive statistics directly on the pathways without using DEGs. This approach is more sensitive to a concordant change of expression in the same direction, even if the transcripts would not be otherwise identified as DEGs. While more sensitive to directionally dysregulated pathways than GCs, current implementations of PCs are not designed to identify dysregulated pathways with both upregulated and downregulated transcripts.

However, a limitation when focusing on the identification of DEPs relies on the selection of the considered prior knowledge on pathways. Currently, several knowledge sources, such as KEGG [18], Reactome, [26] and Pathway Common [27] can be used. This may cause redundancy and different results; moreover, such data sources may contain incomplete, incorrect or inconsistent data. Such dependencies between pathways could result in correlated *P*-values and over dispersion of the number of significant pathways, leading to biased results [28]. Therefore, future studies are required to compare the robustness of DEP methods in presence of noise and missing gene set annotations.

While these approaches for transcriptome analysis are strong in the right context and if properly powered, only few are designed to scale down to individuals. For many DEG detection methods, this failure to scale down to a single subject is an inherent limitation of the underlying mathematical constructs, as they rely on a minimum of three replicates to assess gene-level variance, overdispersion and/or other parameters requiring multiple subjects. Under most experimental designs, cross-sample replicates are used, though triplicate samples from the same individual could potentially be used as a proxy when these are not resource limiting. Although the cost of high-throughput sequencing has been declining, it is still resource-prohibitive to sequence multiple samples, especially when sample procurement is naturally invasive.

Other conventional approaches for analyzing transcriptomes exploit curated knowledge of a particular disease to specifically examine validated or hypothesized markers whose gene expression differs from the reference ‘normal’ or is expressed above a predetermined threshold. This is the case for Oncotype DX™ [29], PAM50 [30] and other clinically available tests that classify samples into tumor subtypes. Reliance on a predefined panel of genes dodges the problems of dimensionality and signal-to-noise detection in raw transcriptome data, but limits scalability across multiple characteristics of a disease and prevents the investigation of novel transcripts and disease mechanisms. To address these issues, other clustering-based techniques can be applied to gather patterned genes across/within samples or data sets (for a review, see [31]) and to obtain classifiers which can then be explored for within-group commonalities and cross-group differences [32]. However, they require a large number of samples, as well as careful external validation in large data sets that have adequate protection from bias and have been reviewed elsewhere [33].

### Single-subject transcriptome analyses

In the context of SSA, several studies have been proposed for extracting relevant biological knowledge from transcriptome data without the large cohort requirement. These approaches can be divided into different categories based on either (i) the number of samples from the same subject they require or (ii) the type of output they provide.

**Figure 3:**
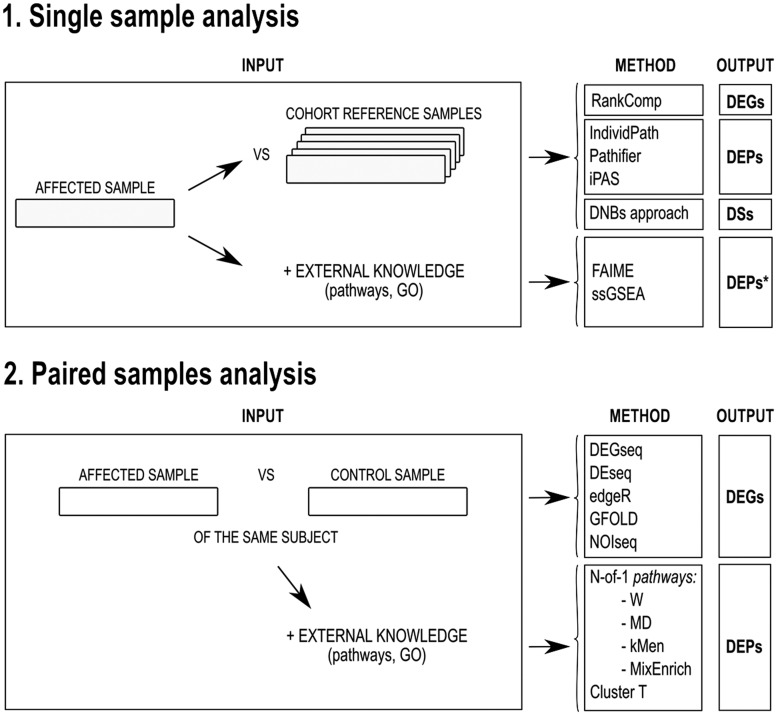
Current strategies to analyze single-subject transcriptomes. Analysis of single-subject transcriptome can be usually divided into two categories based on the required number of samples: (i) single sample analyses, (ii) paired sample analyses, or (iii) more samples (not shown). They can also be categorized according to their outputs: (i) Differentially Expressed Genes (DEGs), (ii) Differentially Expressed Pathways (DEPs), or Disease Scores (DSs). *Note*: DEP* = not true DEP, rather a relative expression level of the pathways because there are no references or baseline to compare the pathway expression of a single sample.

**Figure 4:**
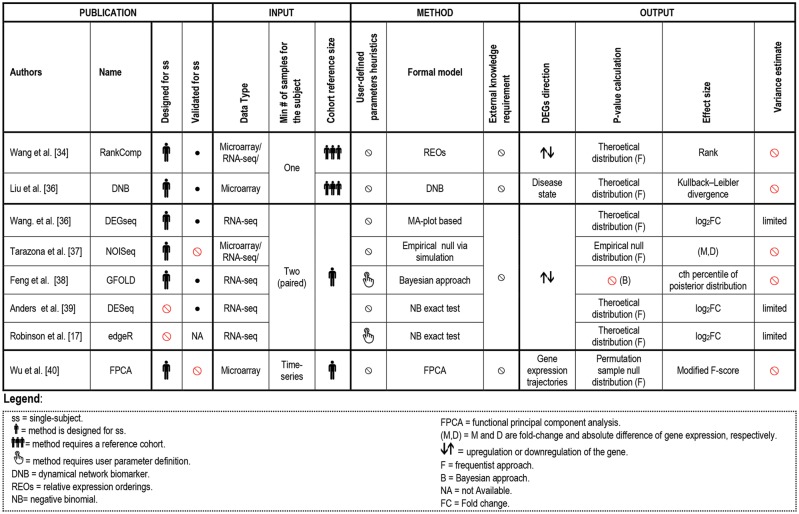
Summary of single-subject methods that analyze transcriptome data to identify DEGs. *Note:* Additional details are available in Table 2.

**Figure 5:**
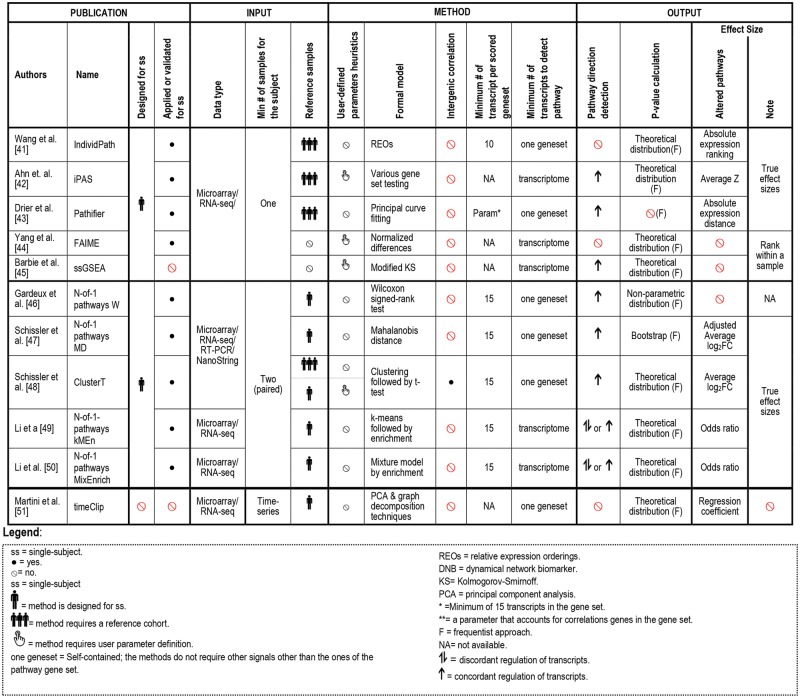
Summary of single-subject methods that analyze transcriptome data to identify DEPs.

As illustrated in Figure 3, single-subject studies can be categorized into GC (DEGs; Figure 4 and Table 2) or PC (DEPs; Figure 5). Based on this classification, we reported the related studies in ‘DEGs identification in single subjects’ and ‘DEP identification in single subjects’ sections, respectively.

**Table 3:** Additional details on single-subject transcriptome analyses of DEPs

Publication	Name	Description
Wang *et al.* [41]	IndividPath	IndividPath computes REOs from a pathway point of view reducing the dimension of the sample representation. Patient-specific DEPs of a sample are obtained by applying a similar procedure to RankComp [35], in which REOs in an individual sample are compared with the highly stable REOs identified from a large cohort of normal samples. The authors identify the biological pathways with significantly disrupted ordering of gene expression via *P*-values. In this case, *P*-values are determined by testing whether the frequency of reversal gene pairs observed in a sample within each pathway is significantly greater than that expected by chance using the hypergeometric distribution model (i.e. a Fisher’s exact test)
Drier *et al.* [43]	Pathifier	Pathifier has been developed to compute PDSs for cancer tumor samples by aggregating gene-level information into pathway-level information, providing meaningful dimension reduction. Pathifier analyzes one pathway at a time and assigns a PDS to each sample by using the expression levels of the genes belonging to the pathway. To calculate PDSs, a PCA is performed to reduce the dimensions and capture the variation of the data. Next, the method identifies the best principal curve using both cohort samples (normal and disease). Then, the PDS of a sample is obtained by computing the distance of a single sample from the median of the normal samples on the principal curve. The output of this approach is therefore a list of DEPs for each sample representing the level of deregulation of each pathway
Ahn *et al.* [42]	iPAS	iPAS provide gene-level statistics (i.e. Z-score) by standardizing the gene expression level of the disease sample with the mean and the standard deviation of the normal samples. Z-scores are used as inputs to calculate iPAS for the disease sample, for example, using the average of the Z-scores in a pathway. iPAS is then computed for every normal sample to construct a null distribution, which assesses the significance of disease iPAS’s deviation from the normal reference.
Yang *et al.* [44]	FAIME	The FAIME transforms a vector of mRNA quantification into pathway-level metrics derived from a single biological sample. Each mRNA is annotated to a gene, and genes are annotated to gene sets via knowledge base integration. Every pathway receives a score that quantifies the ‘average’ over-expression of genes within the pathway, when compared with genes in background (not in the pathway). This process provides mechanism-level interpretation to a single transcriptome.
Barbie *et al.* [45]	ssGSEA	ssGSEA uses the difference in empirical cumulative distribution functions of gene expression ranks inside and outside a gene set (i.e. pathway) to calculate an enrichment statistic per sample, akin to the FAIME methodology described above. The procedure adopted is similar to GSEA [21] except that ssGSEA uses gene expression intensity at the single sample level to compute enrichment scores
Gardeux *et al.* [46]	N-of-1 pathways Wilcoxon	This method aggregates gene expression values from two paried samples into gene sets provided by external knowledge sources (e.g. GO, KEGG). Each externally defined gene set is assessed for differential expression using the nonparametric analog of a paired *t*-test, the Wilcoxon signed-rank test. The result is a metric of pathway-level dysregulation in the form of either a *P*-value or corresponding signed z-score (sign indicates whether the case sample is upregulated or downregulated compared with baseline sample). Computing such a metric across all pathways in an ontology provides a mechanistically anchored profile of personal transcriptome dysregulation for each patient
Schissler *et al.* [47]	N-of-1 pathways MD	N-of-1-pathways MD seeks to improve the differential expression testing component of the framework introduced by Gardeux *et al.* [58]. The rationale behind using the statistical generalization of distance is to incorporate the observed covariance structure between the two paired samples (as they are derived from the same patient). Briefly, the average log2 fold-change of expression within the pathway is adjusted using components of the variance–covariance matrix. Then, a nonparametric bootstrap is performed to estimate the standard error of the pathway average expression. This provides pathway metrics that are more clinically relevant than a Wilcoxon test statistic and simulation studies showed increased power under the MD framework
Schissler *et al.* [48]	ClusterT	The Cluster-T is yet another improvement to the differential test procedure of N-of-1-pathways. It was shown that under nontrivial inter-genetic correlation, the bootstrapping procedure of the MD failed to produce adequate estimates of the standard error of the average log2 fold-change of expression. This problem proved to be challenging without bringing in external knowledge of context-specific gene–gene correlation. With this external knowledge, genes are clustered within pathways and, under certain assumptions, the test statistic was shown to follow a *t*-distribution with degrees of freedom dependent on the number of clusters. In novel multivariate gene expression simulations, the Clustered-T showed far superior performance in false-positive rates
Li *et al.* [50]	N-of-1-pathways MixEnrich	N-of-1 pathways MixEnrich improves both N-of-1 pathways Wilcoxon and MD by detecting DEPs when they are bidirectionally dysregulated and/or background noise is present. Both Wilcoxon and MD are not designed to detect dysregulated pathways with upregulated and downregulated genes (bidirectional dysregulation), which are ubiquitous in biological systems. MixEnrich identifies bidirectional dysregulation by first clustering genes into upregulated, downregulated and unaltered genes. Subsequently, MixEnrich identifies pathways enriched with upregulated and/or downregulated transcripts. The enrichment test performed by MixEnrich detects only pathways with a significantly higher proportion of dysregulated genes with respect to the background. It is therefore more robust in presence of background noise (i.e. a large number of dysregulated genes unrelated to the phenotype)
Li *et al.* [49]	N-of-1-pathways kMEn	N-of-1 pathways kMEn further improves the N-of-1 pathways MixEnrich method by using a nonparametric model (i.e. *k*-means clustering) to cluster genes into upregulated, downregulated and unaltered clusters. The distribution of log2 fold-change of gene expression is complex and may vary from experiment to experiment. Hence, a nonparametric model might be more flexible to model that distribution

REOs = Relative expression orderings.

**Table 4:** Summary of the method validation in single subjects

Publication	Method	In silico validation	Real dataset validation	Independent dataset validation	*In vitro* validation	*In vivo* validation	Clinical trial validation
Transcriptome							
Gardeux *et al.* [46]	N-of-1 pathways W	•	•	⊘	•	⊘	•
Wang *et al.* [36]	DEGseq	•	•	•	⊘	⊘	⊘
Anders *et al.* [39]	DESeq	•	•	•	⊘	⊘	⊘
Feng *et al.* [38]	GFOLD	•	•	•	⊘	⊘	⊘
Wang *et al.* [34]	RankComp	•	•	•	⊘	⊘	⊘
Yang *et al.* [44]	FAIME	•	•	•	⊘	⊘	⊘
Drier *et al.* [43]	Pathifier	⊘	•	•	⊘	⊘	⊘
Li *et al.* [50]	N-of-1 pathways MixEnrich	•	•	⊘	⊘	⊘	⊘
Li *et al.* [49]	N-of-1-*pathways* kMEn	•	•	⊘	⊘	⊘	⊘
Schissler *et al.* [47]	N-of-1 pathways MD	•	•	⊘	⊘	⊘	⊘
Liu *et al.* [35]	DNB	•	•	⊘	⊘	⊘	⊘
Wang *et al.* [41]	IndividPath	⊘	•	⊘	⊘	⊘	⊘
Ahn *et al.* [42]	iPAS	⊘	•	⊘	⊘	⊘	⊘
Schissler *et al.* [48]	ClusterT	•	⊘	⊘	⊘	⊘	⊘
Tarazona *et al.* [37]	NOISeq	⊘	⊘	⊘	⊘	⊘	⊘
Robinson *et al.* [17]	edgeR	⊘	⊘	⊘	⊘	⊘	⊘
Wu *et al.* [40]	FPCA	⊘	⊘	⊘	⊘	⊘	⊘
Barbie *et al.* [45]	ssGSEA	⊘	⊘	⊘	⊘	⊘	⊘
Martini *et al.* [51]	timeClip	⊘	⊘	⊘	⊘	⊘	⊘
Multi-omics							
Vaske *et al.* [69]	PARADIGM	•	•	⊘	⊘	⊘	⊘
Chen *et al.* [68]	iPOP	⊘	•	⊘	⊘	⊘	⊘

**Figure 6:**
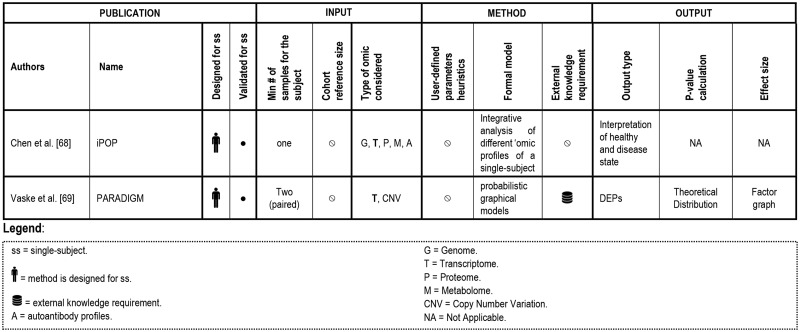
Summary of single-subject methods that analyze transcriptome data combined with other -omics.

The selected studies can be further divided according to the number of samples involved: (i) analysis of single samples (top of Figure 3), (ii) analyses of single individuals using paired samples from the same subject (bottom of Figure 3) and (iii) multiple measurements in single subjects (not shown in Figure 3). This last class of methods is reported in ‘Longitudinal time series analyses of transcriptome’ section.

The utility of single-subject discovery of differentially expressed patterns is central to precision medicine. For example, implicating DEGs in a patient may identify an unconventional treatment (i.e. personal drug repositioning) for this disease, assuming that these DEGs are well-established targets of the drug [52] in another related disease state (e.g. cancer targets). On the other hand, if the aim of the study is to investigate a disease or a particular condition from a broader point of view and to promote greater interpretation of the gene expression results, DEP analyses should be preferred. For example, Figure 5 refers to methods directly imputing DEPs from the transcriptome, i.e. PC approaches. To the best of our knowledge, these methods have not been compared with enriching DEGs into DEPs using methods from Figure 4. However, these DEP methods have been evaluated *in vitro* and *in vivo* as shown in the last section of the article, thus remain the validated strategy for imputing DEPs until properly compared with single-subject DEGs followed by enrichment.

Another key difference in the analysis of single-subject transcriptome is the number of samples required from the subject. In addition, we have found that successful single sample-methods require not only the individual sample but also a cohort reference (‘reference-based’) to perform comparisons and to detect DEGs or DEPs (Figures 4 and 5, column ‘Cohort reference size’). This type of strategy is particularly useful when matched normal and disease samples are unavailable or limited (e.g. brain or heart tissue samples).

Analysis of individuals using paired samples naturally requires that both samples be drawn from the same subject. As samples are isogenic aside from potential somatic variation and/or taken from the same tissue and environmental context aside from any experimentally induced stimuli, this design increases the signal-to-noise ratio and improves the detection of relevant DEGs or pathways. For example, studying both tumor and non-tumor tissues from a cancer patient focuses attention on pathogenic and compensatory mechanisms that differentiate the two tissues because of the disease state.

While we review the methods that strive to mine the most information from limited data (e.g. a pair of transcriptomes of a single subject), investigators need to be cautious that methods do not replace data [53].

An additional aspect we underlined is the requirement of user-defined parameters heuristics of the considered publications heuristics (Column 8 in Figures 4 and 5). Automated methods, not requiring user-defined parameters, are considered superior, as they are less biased and more convenient.

The following subsections will focus on methods for imputing single-subject DEGs (‘DEGs identification in single subjects’ section) and then single-subject DEPs (‘DEP identification in single subjects’ section).

### DEGs identification in single subjects

In this section, we outline and describe emerging studies aimed at extracting DEGs starting from: (i) one sample of the individual and (ii) paired samples drawn from the same subject. A detailed description of the methods is provided in Figure 4 and Table 2.

#### Approaches based on a single sample of an individual

We identified two single-sample methods ([34, 35]) designed to inform on an individual’s transcriptome aberrations. Both methods require the application of a cohort reference, but differ in their predicted outputs. The first one, called RankComp [34], identifies DEGs by comparing the gene expression of the affected sample with a baseline, akin to a reference genome or normal range for clinical testing. RankComp has been applied separately to both total mRNA and microRNA (miRNA) investigations [54]. In the second study, they demonstrated the power of their method to identify deregulation of miRNAs and miRNA–target pairs with mutually exclusive alterations. This approach has the limitation of not being sensitive enough for detecting genes whose differentially expression causes minor changes in the ranking. The second method [35], DNB (Figure 4), different from RankComp, predicts critical disease transition from one sample of an individual, by comparing it with multiple control samples (from other data sets). This type of approach is particularly interesting for investigating individual profiles and classifying them as healthy, pre-disease or disease state.

#### Approaches based on paired samples of an individual

Although DEG identification often requires a large cohort of samples, a few attempts have been made to identify DEGs from only a pair of transcriptomes. These methods provide an opportunity to identify a set of personalized DEGs of a single subject without requiring costly transcriptome replicates. Among these methods, DESeq [39] and edgeR [17] were originally designed as cohort-based methods (Figure 4, column ‘Designed for ss’), but have wide applications. When replicates are not available, these two methods can still be applied. Without replicates, DESeq is conservative, as it assumes the majority of the genes as non-DEGs and estimates a mean–variance relationship from treating the two samples as if they were replicates. edgeR’s performance relies on investigators’ understanding of study, as a parameter in the model is predetermined by the biological nature of the samples. DEGseq [36] is designed for discovering DEGs from only a pair of transcriptomes; yet, its assumption of binomial distribution of RNA-Seq data is insufficient when overdispersion in gene expression is present. NOISeq-sim [37] simulates replicates when real replicates do not exist. With the simulated replicates, NOISeq-sim generates a joint null distribution of fold-changes (M) and absolute differences (D) of the expression counts from the replicates within the same condition. This joint null distribution is then used to assess differential expression by a gene’s (M, D) pair computed between conditions. Finally, GFOLD [38] is another method designed for transcriptome analysis without replicates, as it provides biologically meaningful gene ranks of differential expression, but no significance assessment.

### DEPs identification in single subjects

In this subsection, we report other methods that create biologically interpretable results from a single subject’s transcriptome bypassing detection of significant differences in gene-level expression to go directly to pathway-level signals (DEPs). Such analyses aim to promote a higher-level interpretation of the underlying gene expression data, providing a holistic view of pathway perturbation, instead of focusing attention on any particular gene. All the approaches belonging to this category incorporate a large body of prior biological knowledge (e.g. pathway knowledge sources such as KEGG [18, 55]). This allows researchers to reduce the dimension of a transcriptome-wide gene list (∼22k in human) to a much smaller set (e.g. ∼5000 GO-BP terms) which is then analyzed according to term or pathway overrepresentation or other involvement. This dimension reduction has been showed to improve the prediction of prognosis and therapies [56, 57]. A detailed description of the methods is provided in Figure 5 and Table 3.

#### Approaches based on a single sample of an individual

We identified three methods that require a single sample of an individual and a cohort reference (individPath [41], Pathifier [43], individualized pathway aberrance score, iPAS [42]) and two approaches capable of extracting DEPs from within an individual’s transcriptome without external comparison (single-subject GSEA, ssGSEA [45], Functional Analysis of Individual Microarray Expression, FAIME [44]) (Figure 5). A detailed description of the methodologies used by these studies is provided in Table 3.

Each of the reference-based methods begins by aggregating gene-level information into pathway-level information, providing meaningful dimension reduction, and then apply statistical analyses directly at the pathway level. The first method, individPath, uses relative expressions orderings to directly stratify patients based on individual deregulated pathway status. The authors showed that individPath could predict individually identified, but in-common pathway biomarkers from lung adenocarcinoma and breast cancer data sets that were correlated with survival analysis.

The second method is Pathifier, which computes pathway deregulation scores (PDSs—Table 3) for SSA using principal component analysis (PCA) and curve fitting. Drier *et al.* [43] showed how PDSs successfully reflect deregulation of pathways in glioblastoma and colorectal cancer data sets and could provide clinically relevant stratification of patients. Pathifier has also been successfully applied to provide a classification of breast cancer subtype [59] and to perform a personalized analysis for understanding the status of homologous recombination pathway dysregulation in breast cancer [60].

Finally, an additional method proposed by Ahn *et al.* [42] quantifies the aberrance of an individual sample’s pathways by comparison with accumulated normal data. The authors provide gene-level statistics (i.e. Z-score) by standardizing the gene expression level of the disease sample with the mean and the SD of the normal samples.

DEP approaches requiring a cohort reference, such as iPAS, Pathifier and individPath, are constrained by (i) the number of available normal samples (power), (ii) platform-dependencies and (iii) limited sensitivity to detect pathways that contain only few genes. The large number of normal cohort sample required limits the applicability of these methods in infrequent diseases, or when obtaining appropriate samples and/or defining an appropriate ‘normal’ state is complex. Moreover, the reference cohort may be heterogeneous, and pooling together normal samples means that transcriptome of different individuals is merged, which can obscure stratification and correlation patterns in the normal data.

Because of these limitations, other methods have been proposed to circumvent the normal reference requirement using solely a sample from the subject under study. These strategies aim to reduce dimension by injecting domain knowledge while reducing gene-level noise inherent to a single case sample. Two such methods are the FAIME [44] and ssGSEA [45]. Both methods seek to quantify the effect size and statistical significance of consistent overexpression or underexpression of aggregated gene expression within externally defined gene sets, compared with the genes not annotated to the gene set (background). In the terminology of Goeman and Buhlmann [61], this framework is ‘competitive’ in the sense that scores reflect relative gene set expression when compared with the background. In this manner, both FAIME and ssGSEA detect aberrant pathway expression for an individual’s sample. The methods differ in implementation, however; FAIME operates on the normalized gene expression, while ssGSEA performs calculations on the ranks.

A limitation of these two methods is that they provide a ranking of pathways in terms of their deregulated with respect to other pathways using the gene expression data of the individual (e.g. more or less expressed than an average expression). Therefore, these methods do not identify functionally altered pathways against a reference as in the previous methods because a pathway more or less expressed than average may the normal expected level of expression of that pathway.

The output of both ssGSEA and FAIME report DEPs, which allow enhanced functional interpretation of disease-associated biological processes relative to less readily interpretable lengthy lists of DEGs. This approach could be useful when little pathological knowledge is available for the disease or when substantial pathway heterogeneity may underlie the clinical phenotype. In the case of single-subject studies, DEP lists can be used not only to investigate biological mechanisms specific of certain patients but also to suggest potential treatments or combination of treatments based on gene products annotated to the pathology-associated DEP, or other known interactions.

#### Approaches based on paired samples of an individual

In the following, we will focus on single-subject-based methods that analyze paired samples without the requirement of replicates. In this category, we identified the methods known as N-of-1-pathways (Figure 5 and Table 3). These methods provide a statistical informatic approach by aggregating gene-level measurements from two samples into gene sets (pathways) provided by curated knowledge bases (e.g. GO, KEGG). This consolidation seeks to reduce noise from gene-level measurements and provide meaningful dimension reduction. These profiles are designed to have clinical translational value by providing a systems biology perspective instead of focusing on single biomarkers. A drug targeting a non-DEG product at first glance could seem useless. However, the pathway could yet to be dysregulated and the drug may still have therapeutic value. For example, an epithelial growth factor receptor inhibitor (erlotinib) was successfully used in dual therapy to abate pathway-wide overexpression in oral carcinomas [58].

The first transcriptome analytic framework for quantifying within-patient differential expression from a pair of samples was introduced by Gardeux *et al.* [46] that developed the N-of-1-pathways Wilcoxon. Schissler *et al.* [47] extended the analysis of within-patient paired samples with the N-of-1-pathways Mahalanobis distance (MD) to improve on the Wilcoxon-based approach. MD provides an effect size of pathway-differential expression that incorporates the variance–covariance structure between the two samples. However, MD’s testing procedure failed to account for inter-gene correlation within pathways, which could result in the inflation of false-positive rates [48]. In response to this shortcoming, Schissler *et al.* [48] developed ClusterT to estimate co-expression of genes within the relevant biological context. For example, TCGA breast cancer RNA-Seq samples could be used to characterize clusters of genes within pathways, with positively correlated genes within the same cluster. This approach bears similarities to the ‘accumulated normal sample’ strategy described above, but differs in the way that an ontology is characterized by clusters within the context of analyzing a single subject’s pair. The authors envision co-expression cluster-augmented knowledge bases to enable clinical translation without the additional burden of accumulating *ad hoc* normal samples. N-of-1 pathway Wilcoxon, MD and the Clustered-*T* approaches perform gene set testing, one pathway at a time, in a ‘self-contained’ fashion [61]. This offers an opportunity for small-scale gene expression testing, as whole-transcriptome measurement is not required.

Seeking gene set test procedures in the paired-sample setting that explore pathway-level expression relative to the rest of the transcriptome (i.e. a ‘competitive’ test), Li *et al.* developed two procedures, N-of-1-pathways *k*-Means Enrichment (kMEn) [49] and Mixture-Enrichment (MixEnrich) [50]. The benefits of the techniques lie in the detection of bidirectional pathway dysregulation (mRNAs within the same pathway that are both overexpressed and underexpressed) and in noisy samples with a high frequency of DEGs.

All the N-of-1 pathways methods showed their power in the identification of DEPs that result from diverse health disorder [46, 47–50].

### Longitudinal time series analyses of transcriptome

Biological processes are highly dynamic, and understanding how diseases evolve over time can reveal factors involved in determining the disease status, progression and compensation. However, -omic technologies are typically gathered at infrequent or even single static points. Comprehensive longitudinal -omics data (i.e. one or more type of omics measured over time) can provide key information for understanding the whole evolution of biological processes and underlying biological mechanisms. Comprehensive longitudinal analyses are typically limited by the substantial associated costs of sample collection and patient follow-up. As a result, with perhaps rare exceptions, long time series experiments have few or no isogenic replicates in single subjects. Traditional analysis of time series gene expression data aims at identifying gene sets that exhibit common or distinct patterns of expression between two or more conditions (i.e. gene modification, treatment). The computational complexity to analyze such data is higher, as time course data involves the three dimensions of gene, time and condition. When considering time series data from an individual, several strategies can be applied depending on the experimental setup (i.e. number of time points and condition considered). For example, baseline comparisons can be performed by considering samples gathered during ostensibly healthy physiological states of the patient as the reference population if multiple time points are sampled. Samples gathered during ostensibly healthy physiological states of the patient approaches to extract meaningful knowledge from time series transcriptome data are based on clustering algorithms [62], hidden Markov models [63], Gaussian processes [64] or Bayesian approaches [65] (for a review, see [66, 67]). These techniques can be applied for single-subject transcriptome analysis to extract DEGs or gene expression trajectory patterns from multiple experimental conditions where multiple time points are studied.

However, when replicates are not available, few models have been proposed for the identification of DEGs or DEPs from longitudinal data. In Figure 4, we reported a method [40] aimed at extracting DEGs from time series data, i.e. gene whose expression changed significantly with respect to time. Wu *et al.* [40] propose a nonparametric method that integrates a functional principal component analysis (FPCA) into a hypothesis testing framework to extract gene-specific expression trajectories. As this approach is based on FPCA, the user has to select the number of the first principal components that can explain the data. Therefore, the selection of such parameters can affect the overall results. On the other hand, Martini *et al.* [51] developed an approach to extract time-dependent pathways (DEPs) without the requirement of replicates (Figure 5). This method combines dimension reduction and graph decomposition theory. It first extracts time-dependent pathways and decomposes them into cliques to isolate the time-dependent portion. Although this approach is tailored to time course gene expression data without replicates, it does not provide information about the directionality of the identified DEPs. Its output is the activation versus non-activation of a pathway. Moreover, it has been designed specifically for long time series data, not showing statistical power with short time course data. Therefore, it cannot be applied to investigate biological processes involving small time series (generally short-term) responses.

One of the major limitations of all transcriptome analyses is their inability to fully capture the dynamics of the represented system because of, for instance, posttranscriptional modifications. To this end, analysis of the product of transcriptome can provide significant insights and source of information.

## Single-subject transcriptome integrated with other -omics

In this section, we will report the state of art of current analyses aimed at analyzing the transcriptome combined with other -omics for SSA. The retrieved studies are summarized in Figure 6.

The integration and analysis of different high-throughput molecular assays and data is one of the major topics in precision medicine for understanding patient-specific variations. This approach enables the possibility of obtaining a comprehensive view of the genetic, biochemical, metabolic, proteomic and epigenetic processes underlying a disease that, otherwise, could not be fully investigated by using single -omics approaches. The increased power of multi-omics studies have been already assessed in the understanding of diseases, biomarkers and drug discovery. These methods are based on supervised or unsupervised machine learning techniques and typically aim at classifying patients into cancer subtypes [70–74] or are designed for drug repurposing [73, 75, 76]. Even if these strategies are useful for precision medicine, they are not able to extract meaningful knowledge on individual-specific biological mechanisms. They still rely on the integration of -omics profiles from populations of subjects.

In our review of the literature, only few computational single-subject algorithms aimed at analyzing transcriptome data combined with other -omics have been proposed in the past years. Chen *et al.* [77] pioneered an ambitious project to integrate, analyze and provide clinically interpretable results from multi-omics profiles of an individual. The authors proposed the integrative personal -omic profile (iPOP), using Dr Snyder’s -omics as a test case colloquially referred to as the ‘Snyderome’. iPOP combines genomic, transcriptomic, proteomic, metabolomics and autoantibody profiles collected from a single subject over a 14-month period. A key aspect of this study, other than the focus on collecting data from a single person, was its comprehensive longitudinal nature and sampling during a variety of incidental environmental exposures including two viral infections and physician-recommended diet changes. This resulted in 3 billion measurements taken over 20 time points and >30 TB of data [77]. The article confirmed that some disease risks could be assessed from the genome sequence of the patient, but actual onset and assessment of certain other diseases, such as hypertriglyceridemia, could not be diagnosed based only on the genomic profile. Interestingly, proteome and metabolome were also required to understand the biological mechanisms underlying response to the viral infections. Association between expression and disease status was also revealed through the analysis of transcriptome data.

PARADIGM [78] integrates transcriptome and DNA CNV data to compute pathway scores that represent the alternation of a person’s pathways. Pathway scores are calculated as a joint probability of a directed factor graph, a form a probabilistic graphical model. Variables in the graphical model correspond to different molecular entities; edges in the graph represent within- or between-scale interactions. The interactions are determined by central dogma and knowledge of annotated pathways, such as pathway interaction database [77].

## Validation of single-subject omics methods

We further classified each publication in this review according to the method(s) used for result validation (Table 4). The majority of approaches have been validated with *in silico* simulation of data or by cross-validation in the same data set. A few methods have validated their results across replicate samples, or have had pathology-associated DEG or DEP results successfully reproduced in independent data set. To our knowledge, only the N-of-1pathways W [46] SSA method has been validated *in vitro* and as a prognostic outcome classifier in a prospective study. In that study, patient-specific DEPs were identified in response to an *ex vivo* stimulation of their PBMCs with rhinovirus and used to accurately predict risk of asthmatic exacerbation in those same patients over a 2-year follow-up period. This strongly supports the conclusion that the field of single-subject studies of personalomes is an emerging field that is in need of more rigorous validations for translation to clinical practice. Additionally, new validation strategies need to be developed for *in vivo* and clinical trial validations of personalome imputations.

## Clinical applications

To better understand the requirement for single-subject studies, we revisit the types of approaches and transformations required for clinicians to interpret the more clinically used method of DNA sequencing. As shown in Figure 1, we highlighted the critical steps for clinical interpretation of DNA sequencing. The full genome of 3.5 billion base pairs is evaluated against reference genomes to identify the single-subject variants and mutations, yielding a substantial dimension reduction as well as a transformation from molecular data to a biomolecular interpretation of the sequence. Additional studies provided external knowledge for clinical interpretation. For example, missense and nonsense mutations are known to affect the host genes, many of which are known to lead to Mendelian diseases annotated in OMIM [78]. Reproducible genome-wide association studies led to the creation of the NHGRI Catalog that annotates the disease risk associated to certain single-nucleotide polymorphisms. In other words, for DNAseq, an SSA (intermediate step of mutation and variant calls) precedes clinical utility studies. However, this has not been the case for the majority of the studies at other omics scales.

This review focuses on comparing and contrasting SSA that incorporates this previously unavailable intermediate step for other molecules of life, such as mRNAs, miRNAs, proteins, methylated DNA regions and metabolites (carbohydrates and lipids). For example, oncologists already use assays for determining expression fold change and protein function of oncogenes and tumor suppressors through the comparison of tumor tissue with external references or unaffected paired tissue. As these curated approaches may not scale to the full omics data for other diseases, we provide emerging evidence that the newly available unbiased SSA enables new types of studies investigating their clinical utility by addressing the gap of biomolecular interpretation of raw omics signal. Among possible studies, we demonstrate that omics clinical prediction classifiers that operate directly at the omics scale may be redesigned for the parsimonious transformed signal of single-subject studies for improved clinical utility. For example, Gardeux *et al.* [79] quantified the personal pathway-level transcriptomic response of peripheral blood mononucleocytes to rhinovirus *ex vivo* and trained a classifier predictive of children prone to asthma exacerbations. The dimension of the signal was reduced from the entire transcriptomes of paired samples in 20 subjects (∼10^6^ data points) to the effect size of statistically significant responsive pathways in at least one subject (∼10^4^ data points).

While many unbiased fully specified GExpCs designed over the entire transcriptome have been published in peer-reviewed journals, few have been FDA approved because of their lack of a mechanistic relationship between the features (gene transcripts) and the disease progression [6, 80, 81]. Two additional important limitations of the clinical utility of conventional GExpCs include (i) their platform dependence that limits their face-value validity (e.g. specific to AgilentTM) [8], and (ii) distinct GExpCs are paradoxically obtained from distinct cohorts of the same phenotypes [6, 8]. Interestingly, the transformation of a signal from conventional raw gene expression to effect size obtained after DEP-type SSA enables us to address these three limitations. First, SSA generates an effect size and *P*-value for each subject, analogous to mechanisms-level features ascribed to a patient. In addition, Zhang *et al.* [44] have shown that the FAIME DEP transformation leads to the rediscovery of at least 50% of the same gene set-level features (KEGG, GO) in seven distinct data sets of head and neck cancers when learning fully specified gene set-level classifiers (GenesetCs). The discovered features were consistently predictive of disease progression in independent validation data sets. Furthermore, three studies demonstrated that the discovered GenesetCs overlap by >50% of gene set features across expression platforms (Affymetrix, Agilent, RNAseq) [44, 82, 83], thus addressing another limitation of GExpC. Finally, a recent report from Gardeux *et al.* [79] shows that DEP single-subject studies in paired samples could generate features of higher quality than those obtained directly from gene expression in small cohorts. Specifically, a GenesetC predictive of exacerbation of pediatric asthmatic patients was confirmed in an independent cohort (learning set 40 subjects, validation set 22 subjects). This study suggests that SSA could reduce the cohort size for classifier development, as conventional GExpCs generally require hundreds of subjects in their learning sets.

## Perspective and conclusion

The development and analysis of personal transcriptome interpretation are essential for precision medicine, as therapeutic decision-making pertains not exclusively to genomic sequences but to Genome x Environment interactions (GxE) as well. For example, isogenic twins may experience different diseases because of their distinct environment exposures, despite sharing identical genomes. Even in the presence of the same diseases, their therapeutic responses may vary as a result of other GxE conditions [79, 84]. The analysis of single-subject transcriptomes is valuable for extracting useful knowledge to better understand individual variability and patient-specific mechanisms underlying a disease and for suggesting tailored therapies. Selecting the best method for evaluation of a given subject’s personalome is first dependent on the biological question and experimental design approach that is best suited for determining an answer.

This review revealed that ongoing advances in high-throughput technologies, emerging research and clinical questions urge continued investigation and development toward experimentally validated methods for unveiling tailored treatments from patient-specific transcriptomes (Table 4). In recent years, this nascent field of single-subject -omics has demonstrated considerable growth as reflected by the number of approaches being published and underlined by the high number of citations for the earlier works (Figure 2). Figures 4, 5 and 6 detail the computational analysis options that are available for transcriptome data and the sampling regimens that each requires to be applied effectively (e.g. single sample, paired samples, longitudinal samples), as well as whether access to an appropriate external reference database is necessary or what type of output is provided (e.g. DEGs or DEPs). Each broad category of currently available methods has both advantages and limitations.

Approximately half of the bioinformatics methods we surveyed perform a comparison between the single subject’s profile and a reference, most often a cohort of accumulated normal samples or samples of a well-defined disease subtype [34, 35, 41–43]. These methods are generally able to capture patient variability and provide clinically interpretable results. However, accumulating the reference may be challenging and not factor in the heterogeneity of the reference sample, and subtle effects may be difficult to detect. This may result in missing crucial alterations present in the patient profiles. Nonetheless, these methods are appropriate when a robust reference is obtainable, and/or cases where a paired sample design does not make sense.

### Recommended DEG and DEP approaches to SSAs

As transcriptomes vary by cell type and with environmental exposures, clinically or biologically interpretable altered mechanisms are more convincing when developed in isogenic (same subject) conditions than in heterogenic ones. We thus recommend clinical or experimental designs that generate a baseline in the same individual, i.e. paired samples (Figures 1, 2 and 5), which are well evaluated (Table 4) and have been validated in many publications (Figure 2). At this point in time, multi-omics methods have not been evaluated sufficiently to recommend one over another, even though they have the potential for being the best methods. Among analytical techniques exploiting a baseline, the more measures the better; thus FPCA and timeClip are favored for DEGs and DEPs, respectfully, when three or more samples are available over time. For discovery of DEGs among paired samples analyses, we recommend the use of DEGseq, as it is designed for single subjects, provides effect sizes and *P*-values, considers a limited variance estimate and is validated in independent samples. On the other hand, edgeR and GFOLD are suboptimal as they require user-defined parameters (Figure 4, column ‘User-defined parameters heuristics’). The unbiased and parameter-free DEseq approach, which is not designed for a single subject, is likely performing better in these conditions than either edgeR or GFOLD that require subjective, and possibly biased, user-defined parameters. However, currently no study has yet been conducted to compare the accuracy of different single-subject DEG methods against one another. For discovery of DEPs in paired samples, N-of-1-pathways kMen has been shown in simulation and in real data sets to outperform other paired DEPs methods; however, the N-o1-pathways Wilcoxon remains the most validated, which includes a clinical trial. Of note, ClusterT is the only approach controlling for intergenic correlation (Figure 5, column ‘Intergenic correlation’) that can create enrichment biases; however, additional validations are required.

In absence of multiple samples from a single subject with its own isogenic reference, we recommend analyses providing biologically and clinically interpretable results of altered expression against heterogenic references (a population). Among single-sample SSA, we recommend RankComp and individPath for DEG and DEP determination, respectively. RankComp is currently the only method that provides DEGs based on the comparison of a single sample against a reference cohort. While for DEP determination, we suggest individPath because of its rigorous formal model and the small number of transcripts required to detect DEPs.

Imputing altered or dysregulated expression of a transcript of a pathway is not feasible for inadequately designed clinical assays or experiments aimed at interpreting a single transcriptome in the absence of any transcriptome reference (e.g. isogenic, heterogenic). To address this, transcript and pathway expression can be compared within a sample using FAIME or ssGSEA. However, the output of higher or lower expression of a mechanism as compared with the sample expression may simply be the normal state of such a mechanism with the interpretation being ambiguous.

While transcriptome analyses can provide DEGs and DEPs for single subjects and are the most mature, we anticipate that as the field advances, it will be possible to reveal novel physiological state correlations through the construction and analysis of multi-scale personalomes. The analysis of a single scale (i.e. -omics data) alone cannot reveal the complex picture underlying a disease that may be fully captured only by fusing together multi-omics data (from genome to metabolome, to exposome) of an individual, i.e. via comprehensive personalome profiles. In fact, the combination of multiple -omics data can lead to the detection of a comprehensive individual variability, essential for providing new insights into disease pathophysiology and mechanisms that may explain the differences in drug responses in the human population. As shown in Figure 1 and discussed in ‘Clinical applications’ section, by delivering dimension reduction and biomolecular interpretations, SSAs enable new types of transcriptomic analyses for clinical interpretation that compare with the methods applied to DNAseq for clinical interpretation. For example, current DNA sequence-based, and classifier-based SSA commercial offerings provide oncologists with annotations of oncogenic or tumor suppressor genes with copy number variants, gain- or loss-of-function mutation, expression fold changes (tumor versus normal) or gene expression against reference tissue and occasionally protein activity. However, these are limited results for a handful of known genes that have been highly curated to apply to a narrow set of diseases, while novel SSA approaches discussed in this manuscript unbiasedly assess the entire transcriptome for DEPs and DEGs in diseases that may be far less well studied, analyses which are not currently available commercially. Clinical utility of these assessments requires additional studies or a knowledge base, similarly to the interpretation of novel mutations for DNA (Figure 1; ‘Studies informing clinical interpretation’).

### Opportunities for future work

Analysis of multi-omics dynamic profiles including transcriptome, proteome, methylome and metabolome can additionally provide indicators of real-time phenotypes and physiology in individuals that cannot be obtained through examination of the static genome alone. In doing so, GxE interactions are revealed [79]. -Omics integration has been used successfully for the identification of novel associations between biological entities (e.g. genes, proteins) and disease [74], patient stratification [73] and biomarker discovery or drug repositioning [76]. However, these strategies have not yet been applied for the integration of multi-omics data of an individual and biological knowledge.

When taking into account the integration of multiple -omics, an important aspect to consider is the variability of data between each -omics, not only with respect to the represented biological process but also with the associated noise levels, identification accuracy, coverage and temporal resolution of data. These differences complicate the integration and joint modeling of multi-omics data. While this is intuitively clear, it remains computationally and experimentally challenging to effectively integrate longitudinal multi-omics data. For one, each biological entity (e.g. gene, metabolites) has different time-dependent modulation and responds to signals on a different specific time scale, even if contributing to the same biological process. Second, biological processes that take place in inaccessible tissues (e.g. brain, internal organs) cannot be feasibly monitored in a longitudinal approach, even if a single sample is possible. Additional challenges are related to the same variables of autocorrelation across repeated measurements, random effects and missing data. Moreover, the design of longitudinal studies of a single subject must account for repeated measures preferably being equally spaced in time, allowing the increase in statistical power of the approach [85]. An obvious opportunity that has not been reported is to learn convergent patterns at one -omics scale (e.g. transcriptome) and correlate it with those of another scale (e.g. proteomics), thus providing internal validation and increasing the noise-to-signal ratio.

Futures studies for SSA of transcriptomes will need to focus on four underreported approaches: (1) variance estimation in isogenic conditions from single-subject measures (without requirement of reference transcriptomes), (2) activity level of pathways (functional, e.g. upregulated versus downregulated) rather than expression direction (overexpressed versus underexpressed), (3) the analysis and integration of comprehensive personal -omics data to infer dysregulated molecules and mechanisms and (4) rigorous validation of DEGs, DEPs, or other results with appropriate *in vitro*, *in vivo* and clinical trial investigations.

As clinical research continues to explore the importance of patient heterogeneity, we encourage more investigators to adopt single-subject study designs and -omics analyses when appropriate to maximize the information made available by high-throughput technologies. Access to these analysis tools may also allow researchers to more thoroughly explore certain rare case studies, outliers and patients-of-special-interest in a way that they could not have done if relying only on traditional large cohort-based statistics. This is particularly true if an isogenic paired-sample study design can be used to answer a meaningful biological or clinical question. The use of personalome integrated with the available external knowledge (e.g. repositories on disease–disease association, target–gene interactions, gene–gene interaction) can provide new opportunities for developing more robust and comprehensive results that account for all the interacting -omics and temporal behaviors of the biological system of an individual.

Finally, personalome researchers should consider a creative application of powerful engineering and mathematical tools that have not yet been applied to study the mechanisms underpinning the personalome of an individual. For example, computational methods used to analyze time series data include generalized linear mixed models, generalized estimating equations, Markov models, nonparametric or semi-parametric models or Bayesian models and dynamic pathway analysis [85]. However, these methods have not yet been applied to study the machinery underpinning the personalome of an individual. Clearly, these methods are not directly applicable as they are cohort-centric; however, innovations may altogether extend the paradigm of their current implementation.

## Supplementary Material

bbx149_Supp

## Notes

Key Points

- For the ‘personalome’ to enable precision medicine from -omics data, we need to move from cohort-focused assays and analytics to individualized (single-subject) studies.
- We survey and categorize methodology by biological and informatics input, mathematical formalism and procedure output.
- Our review focuses on the transcriptome dimension of the personalome showing a great development to date, while proteomics, multi-scale and other scales of biology present open challenges.
- The personalome methods need more rigorous validations, as few have been validated *in vitro*, *in vivo* or in clinical trials.
- The emerging personalome represents a largely unexplored application of -omics data and potentially has important consequences for improving patient outcomes.

## Notes

**Francesca Vitali**, PhD, is a Research Assistant Professor at the University of Arizona. Her main research interests are in pharmacogenomics, drug repurposing, precision medicine, bioinformatics and big data techniques.

## Notes

**Qike Li** is a PhD candidate at the University of Arizona. His research interests are in the area of single-subject analytics with applications in precision medicine.

## Notes

**Grant Schissler** is an Assistant Professor at University of Nevada, Reno. Recently, he has helped to build statistic informatics tools that allow clinical researchers to interpret genomic data of individual patients.

## Notes

**Joanne Berghout**, PhD, is a Research Assistant Professor at the University of Arizona. She uses genetics and ontologies to uncover patterns and candidate genes associated with Mendelian and complex diseases.

## Notes

**Colleen Kenost**, EdD, Director of Operations, Center for Biomedical Informatics and Biostatistic, the University of Arizona. Her role is to translate research prerogatives into action and operationalize strategic plans.

## Notes

**Yves Lussier**, MD, Professor of Medicine and Director, Center for Biomedical Informatics and Biostatistics, University of Arizona. His research group solves problems related to computational precision medicine and translational bioinformatics.
