# Supplementary material for: Developing a ‘personalome’ for precision medicine: emerging methods that compute interpretable effect sizes from single-subject transcriptomes
Source: Brief Bioinform. 2017 Dec 18;20(3):789–805. doi: 10.1093/bib/bbx149 (PMC6585155; doi:10.1093/bib/bbx149)
Supplement: bbx149_Supp [file bbx149_supp.pdf]

Supplement S1 –

Developing a 'personalome' for precision medicine: emerging methods that compute clinically interpretable effect sizes from single-subject omics

Francesca Vitali[1,2,3], Qike Li[1,2,3,4], A. Grant Schissler[1,2,3,4], Joanne Berghout[1,2,3,5], Colleen Kenost[1,2,3] and Yves A. Lussier[1,2,3,4,6, *]
[1]Center for Biomedical Informatics and Biostatistics, The University of Arizona, Tucson, AZ, USA;
[2] BIO5 Institute, The University of Arizona, Tucson, AZ, USA;
[3] Department of Medicine, The University of Arizona, Tucson, AZ, USA;
[4]Graduate Interdisciplinary Program in Statistics, The University of Arizona Tucson, AZ, USA;
[5]The Center for Applied Genetics and Genomics in Medicine, The University of Arizona, Tucson, AZ, USA;
[6]University of Arizona Cancer Center, The University of Arizona
* Corresponding author

Detailed method of the literature survey

We retrieved publications by performing an automated search in PubMed and Google database by using combination of keywords such as single-subject, precision medicine, individual, single sample, patient-specific, personalized omics, transcriptome, multi-omics, computational methods etc. The studies included in this review were journal articles or proceedings written in English. If the same study appears in multiple publications, only the most comprehensive and latest version was included.

We selected relevant studies by first screening titles and abstracts then analyzing full-texts. We included only publications regarding transcriptome or its combination with other 'omics (e.g. proteome, metabolome, methylome, …). For each selected article, we extracted the year of publication and the number of citations. We excluded studies related to (i) genome sequence analysis since the area is already covered.

After reading the selected publications, data from each article was then organized into a matrix table and analyzed with respect to the following criteria: (i) type of 'omics, (ii) the number of repeated measures in a single subject (e.g. paired samples), (iii) the requirement of a reference 'omics set across distinct subjects, (iv) the use of external knowledge base (e.g. genesets from Gene Ontology or signatures), (v) the combination/integration of multiple types of 'omics from a single subject (personalome), and (vi) modeling algorithms (e.g. Bayesian approaches, mixture models, Mahalanobis distance, etc.)

Second, we compared and contrasted these methods through tabular summaries and figures, with focus on the types of (i) data inputs, (ii) outputs (e.g. predictions on genesets/pathways, biomarker discovery), and (iii) type of validation (e.g in silico validation, in vitro validation, in vivo validation, clinical trials).

Third, we highlight the successful approaches for each criterion, and identify promising ones that are either nascent or unexplored as potential opportunities for other applications. We emphasize single-scale and multi-scale methods that improve statistical and interpretative power aimed at reducing knowledge complexity for precision medicine, shifting from a population-based view to a single-subject-based view.
